# Utilization patterns of insulin for patients with type 2 diabetes from national health insurance claims data in South Korea

**DOI:** 10.1371/journal.pone.0210159

**Published:** 2019-03-06

**Authors:** Kyoung Lok Min, Heejo Koo, Jun Jeong Choi, Dae Jung Kim, Min Jung Chang, Euna Han

**Affiliations:** 1 Department of Pharmaceutical Medicine and Regulatory Sciences, Colleges of Medicine and Pharmacy, Yonsei University, Incheon, Republic of Korea; 2 Department of Pharmacy and Yonsei Institute of Pharmaceutical Sciences, College of Pharmacy, Yonsei University, Incheon, Republic of Korea; 3 Department of Endocrinology and Metabolism, Ajou University School of Medicine, Suwon, Republic of Korea; Universidade de Mogi das Cruzes, BRAZIL

## Abstract

Type 2 diabetes mellitus (T2DM) is a chronic disease that requires long-term therapy and regular check-ups to prevent complications. In this study, insurance claim data from the National Health Insurance Service (NHIS) of Korea were used to investigate insulin use in T2DM patients according to the economic status of patients and their access to primary physicians, operationally defined as the frequently used medical care providers at the time of T2DM diagnosis. A total of 91,810 participants were included from the NHIS claims database for the period between 2002 and 2013. The utilization pattern of insulin was set as the dependent variable and classified as one of the following: non-use of antidiabetic drugs, use of oral antidiabetic drugs only, or use of insulin with or without oral antidiabetic drugs. The main independent variables of interest were level of income and access to a frequently-visited physician. Multivariate Cox proportional hazards analysis was performed. Insulin was used by 9,281 patients during the study period, while use was 2.874 times more frequent in the Medical-aid group than in the highest premium group [hazard ratio (HR): 2.874, 95% confidence interval (CI): 2.588–3.192]. Insulin was also used ~50% more often in the patients managed by a frequently-visited physician than in those managed by other healthcare professionals (HR: 1.549, 95% CI: 1.434–1.624). The lag time to starting insulin was shorter when the patients had a low income and no frequently-visited physicians. Patients with a low level of income were more likely to use insulin and to have a shorter lag time from diagnosis to starting insulin. The likelihood of insulin being used was higher when the patients had a frequently-visited physician, particularly if they also had a low level of income. Therefore, the economic statuses of patients should be considered to ensure effective management of T2DM. Utilizing frequently-visited physicians might improve the management of T2DM, particularly for patients with a low income.

## Introduction

Type 2 diabetes mellitus (T2DM) is one of the most prevalent chronic metabolic disorders, typically requiring life-long management. The global number of patients with T2DM increased from 108,000,000 in 1980 to 422,000,000 in 2014, and the prevalence among adults aged ≥ 18 years almost doubled from 4.7% in 1980 to 8.5% in 2014 [1]. Additionally, the number of individuals with diabetes is expected to increase from 425,000,000 in 2017 to 629,000,000 in 2045 globally, and ~90% of the cases are T2DM [2]. In Korea, the financial and societal burdens of T2DM are also substantial; the prevalence rate of diabetes among adults > 30 years was 14.4% in 2016 [3], while the cost of medical therapy increased almost six-fold from 82,500,000 USD in 2002 to 480,000,000 USD in 2013 [4]. Patients with T2DM have a high risk of developing macrovascular and microvascular complications if the disease is not adequately controlled [5]. For example, T2DM is the main cause of chronic kidney disease globally, while diabetic nephropathy develops in 25–40% of patients with diabetes [6,7]. In addition, diabetic neuropathy is the most common cause of neuropathy in developed countries, and up to 50% of patients have neuropathy that causes major complications, including diabetic foot and infection [8–10].

Various oral antidiabetic drugs have been developed to control T2DM. The treatment guideline for T2DM in South Korea is the same as the America Diabetes Association guideline. Guidelines in both Korea and the U.S. diagnose T2DM as follows: 1) HbA1C ≥ 6.5%; 2) fasting plasma glucose ≥126 mg/dL (7.0 mmol/L); 3) 2 h plasma glucose value after 75 g oral glucose ≥ 200 mg/dL (11.1 mmol/L); or 4) diabetic symptom and random plasma glucose ≥ 200 mg/dL [11]. Both guidelines also state that the purpose of treatment of T2DM is to maintain HbA1c < 6.5% [5,12]. The treatment is started with metformin when the HbA1c level is < 7.5%. A second oral antidiabetic agent or insulin can be added if metformin monotherapy is ineffective in 3 months or if Hb1Ac is > 7.5% [5,12–15]. However, insulin can be started as the first treatment when patients with newly diagnosed diabetes have hyperglycemic and metabolic symptoms with HbA1c > 9.0% [15]. In this case, inadequate glucose control usually drives the prescribing of insulin, leading to the assumption that insulin use indicates advanced T2DM [5,12,14,16].

The prevalence of diabetes has increased most rapidly in low- and middle-income countries compared with high-income countries [1]. According to a national diabetes statistics report in the U.S., the prevalence and incidence of T2DM was higher in individuals with lower levels of education, which may be associated with a low income [17]. Studies have also shown poorer health status and lower healthcare usage among individuals on a low income than among those on a high income, indicating that those with a low income may have inadequate access to healthcare services and as a result may be at an increased health risk [18–21]. The relationships between income and diabetes prevalence have been reported in Korea and Asian countries as well [22–27]. However, no studies have been performed to investigate the relationship between income and insulin use in Asia. The present study aimed to assess the use of insulin in patients with newly diagnosed T2DM and to investigate the difference in diabetic management, focusing particularly on the role of their economic status on insulin use.

We also assessed whether access to primary physicians, operationally defined as the frequently used medical care providers at the time of T2DM diagnosis (frequently-visited physician, hereafter) was associated with insulin use. The positive effect of primary physicians in the management of chronic disease, including T2DM, is well recognized in the literature. For example, previous studies in the U.S. have shown that primary physicians had a positive impact on the management of diabetes [28,29]. Interventions by primary physicians have also been reported to improve clinical outcomes in patients with chronic diseases, including hypertension and chronic pain [30,31]. However, no studies on frequently-visited physicians and insulin use have been performed in Korea. The present study may be useful for understanding the implicit role of frequently-visited physicians in the management of diabetes in Korea.

## Materials and methods

### Data

We used insurance claim data from the National Health Insurance Service (NHIS), which provides public health insurance to all legal residents in Korea. All clinics, hospitals, and pharmacies in Korea are required to participate in the NHIS, and they are reimbursed for their services through the NHIS after filing claims electronically. Using these data meant the research was relevant in a real-world context with a nationally representative cohort, providing useful evidence for establishing a national-level management system for T2DM. This study was based on retrospective NHIS data, and all data were anonymized. The study was approved by the Research Ethics Committees of Yonsei University (7001988-201704-HR-175-01E), and informed consent was waived by the IRB.

We retrospectively obtained the data of 1,000,000 respondents, which was approximately 2% of a total random sample from the NHIS data, for the period 2002–2012. During this time, 294,610 patients were diagnosed with T2DM or a diabetes-related disease based on the International Statistical Classification of Diseases and Related Health Problems, 10^th^ revision (ICD-10). The following codes were used to identify target patients: E11 (T2DM), E12 (malnutrition-related diabetes), E13 (other specified diabetes), E14 (unspecified diabetes), G590 (diabetic mononeuropathy), G632 (diabetic polyneuropathy), H280 (diabetic cataract), H360 (diabetic retinopathy), M142 (diabetic arthropathy), N083 (diabetic nephropathy), O24 (gestational diabetes), R73 (hyperglycemia), R81 (glycosuria), and Z131 (special screening test for diabetes).

**Fig 1:**
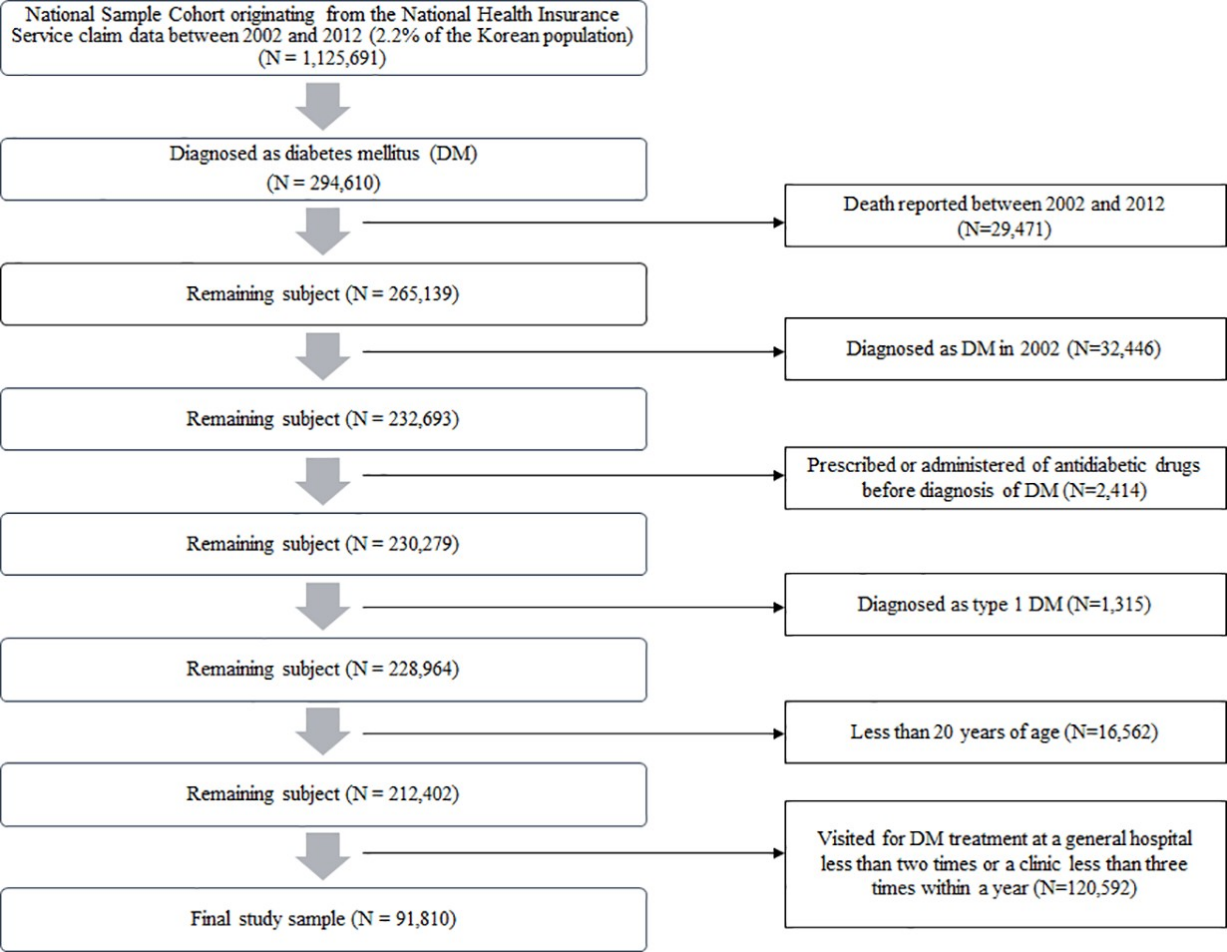
Selection of study sample.

Any patients who succumbed to mortality between 2002 and 2012 (N = 29,471) were excluded. The T2DM codes may have been entered when blood glucose was temporally elevated, however, this does not necessarily mean that the patients are diagnosed as DM. Therefore, various exclusion criteria were applied to reduce false-positives for T2DM. The following exclusion criteria were also applied to restrict the sample to patients who were newly diagnosed with T2DM: 1) diagnosed with DM in 2002 (the initial year of the cohort) (N = 32,446); and 2) prescribed or administered with antidiabetic drugs preceding the diagnosis of DM (N = 2,414). Patients who were diagnosed with type 1 DM (N = 1,315) and were < 20 years old (N = 16,562) were also excluded. Finally, patients who attended hospitals or clinics for DM treatment on fewer than three occasions within a given year were also excluded (N = 120,592). The final sample included 91,810 adult patients (Fig 1). Focusing on patients who were newly diagnosed with T2DM provides a more valid assumption that insulin use for the newly-diagnosed patients implies poor management of T2DM. In addition, as it was not possible to identify when T2DM was diagnosed for patients who were diagnosed with diabetes before baseline in the present study, the underlying reason for insulin use cannot be rightfully assumed [32,33].

### Dependent variable

The pattern of antidiabetic drug use was considered the dependent variable and was defined as follows: 1) non-use of antidiabetic drugs, 2) prescription of oral antidiabetic drugs only, or 3) prescription of insulin with or without oral antidiabetic drugs. Insulin prescription or use was considered to indicate advanced T2DM, consistent with previous studies [5,12–14].

### Independent variables

The main independent variables were health insurance premium and access to a frequently-visited physician. The patients were identified as having a frequently-visited physician if they visited the same clinic or hospital for >90% of all their claims within a 1-year period at the time of T2DM diagnosis. NHIS premiums were classified into five levels, with the reference group as the most affluent. In Korea, this premium is determined based on the earnings and assets of a given household, and naturally there is no differential for healthcare access or out-of-pocket expenses for insured services by premium or income levels. Approximately 4–5% of Koreans are under Medical-aid system, similar to the Medicaid program in the U.S., which waivers premium and the majority of out-of-pocket payments. Patients are able to choose any clinic or general hospital regardless of insurance status. Referral letters are required for first time users of general hospitals, for which the coinsurance rate for out-of-pocket payment is higher than clinics or non-general small hospitals with less than five specialisms [34]. Only for Medical-aid patients clinics are designated when there are risks of too frequent medical visits or drug abuse, and they require a letter from the designated providers to use other clinics or upper level hospitals [35].

The following covariates were controlled for all estimations: age, sex, insurance qualification, prescriber characteristics, Charlson comorbidity index (CCI), residential area, and year of diagnosis. Age was classified into three categories [≤ 34 years, ≥ 35 years but < 65 years, and ≥ 65 years (reference group)]. The insurance qualification was divided into the main beneficiaries and dependents. Prescriber characteristics included type [i.e., general teaching hospitals, general hospitals, and clinics (reference group)] and specialty [i.e., family medicine, others, and internal medicine (reference group)]. The comorbidities of the respondents were weighted to generate the CCI, an index used to predict the estimated 10-year survival rate of patients with various clinical conditions [36]. The CCI score was capped at 4, generating five possible classifications (0, 1, 2, 3, and ≥ 4). The year of diagnosis was generated as a series of dummy indicators.

### Statistical analysis

Multivariate Cox proportional hazards analysis was performed using SAS version 9.4 (SAS Institute, Cary, NC, USA). The hazard ratios (HRs) and 95% of confidence intervals (CIs) were calculated for each variable. The level of significance was set at a p-value of 5%.

## Results

### Summary statistics

The demographic data for the final sample are summarized in Table 1. The results revealed that over half (53.9%) of the sample (91,810 adults) did not use any antidiabetic drugs, whereas approximately one-third (36.3%) used oral antidiabetic drugs and one-tenth (10.1%) used insulin. The sample included marginally more female than male patients, and although the majority of patients were aged 35–64 years, over a third of the sample was aged ≥ 65 years.

**Table 1:** Distribution according to the utilization pattern of antidiabetic drugs^a^.

Variable	Total (N = 91,810)	No antidiabetic drug group (N = 49,151)	Oral antidiabetic drug group (N = 33,378)	With insulin group (N = 9,281)
Sex				
Male	44,604 (48.58)	20,931 (42.59)	18,670 (55.94)	5,003 (53.91)
Female	47,206 (51.42)	28,220 (57.41)	14,708 (44.06)	4,278 (46.09)
Age, years				
20–34	5,671 (6.18)	4,087 (8.32)	937 (2.81)	647 (6.97)
35–64	59,654 (64.98)	29,448 (59.91)	23,957 (71.77)	6,249 (67.33)
≥65	26,485 (28.85)	15,616 (31.77)	8,484 (25.42)	2,385 (25.70)
Health insurance premium levels				
Medical-aid group	5,185 (5.65)	2,504 (5.09)	1,965 (5.89)	716 (7.71)
Levels 1–4	24,823 (27.04)	12,678 (25.79)	9,379 (28.10)	2,766 (29.80)
Levels 5–7	24,425 (26.60)	12,964 (26.38)	8,915 (26.71)	2,546 (27.43)
Levels 8 and 9	22,739 (24.77)	12,534 (25.50)	8,110 (24.30)	2,095 (22.57)
Level 10	14,638 (15.94)	8,471 (17.23)	5,009 (15.01)	1,158 (12.48)
Presence of frequently-visited physicians				
No	78,658 (85.67)	44,327 (90.19)	27,046 (81.03)	7,285 (78.49)
Yes	13,152 (14.33)	4,284 (9.81)	6,332 (18.97)	1,996 (21.15)
Insurance qualification				
Main beneficiaries	46,126 (50.24)	26,361 (53.63)	15,246 (45.68)	4,519 (48.69)
Dependents	45,684 (49.76)	22,790 (46.37)	18,132 (54.32)	4,762 (51.31)
Types of healthcare providers upon diagnosis				
Clinics	50,572 (55.08)	24,391 (49.62)	21,681 (64.96)	4,500 (48.49)
General hospitals	11,348 (12.36)	6,241 (12.70)	3,776 (11.31)	1,331 (14.34)
General teaching hospitals	29,890 (32.56)	18,519 (37.68)	7,921 (23.73)	34,50 (37.17)
Medical specialty of the physicians				
Internal medicine	61,344 (66.82)	30,106 (61.25)	24,888 (74.56)	6,350 (68.42)
Family medicine	6,106 (6.65)	2,654 (5.40)	2,917 (8.74)	535 (5.76)
Others	24,360 (26.53)	16,391 (33.35)	5,573 (16.70)	2,396 (25.82)
Residential area				
Big cities	42,464 (46.25)	22,906 (46.60)	15,393 (46.12)	4,165 (44.88)
Small- and medium-sized cities	39,754 (43.30)	21,253 (43.24)	14,506 (43.46)	3,995 (43.04)
Rural area	9,592 (10.45)	4,992 (10.16)	3,479 (10.42)	1,121 (12.08)
Charlson comorbidity index scores				
0	24,101 (26.25)	15,135 (30.79)	7,154 (21.43)	1,812 (19.52)
1	33,634 (36.63)	18,689 (38.02)	12,154 (36.41)	2,791 (30.07)
2	21,859 (23.81)	10,728 (21.83)	8,733 (26.16)	2,398 (25.84)
3	8,456 (9.21)	3,339 (6.79)	3,696 (11.07)	1,421 (15.31)
≥4	3,760 (4.10)	1,260 (2.56)	1,641 (4.92)	859 (9.26)
Year of diagnosis				
2003	6,278 (6.84)	2,033 (4.14)	2,801 (8.39)	1,444 (15.56)
2004	6,211 (6.77)	2,391 (4.86)	2,706 (8.11)	1,114 (12.00)
2005	7,515 (8.19)	3,364 (6.84)	3,047 (9.13)	1,104 (11.90)
2006	6,219 (6.77)	2,753 (5.60)	2,649 (7.94)	817 (8.80)
2007	7,342 (8.00)	3,585 (7.29)	2,970 (8.90)	787 (8.48)
2008	7,925 (8.63)	3,822 (7.78)	3,218 (9.64)	885 (9.54)
2009	8,462 (9.22)	4,415 (8.98)	3,268 (9.79)	779 (8.39)
2010	8,932 (9.73)	5,114 (10.40)	3,194 (9.57)	624 (6.72)
2011	13,090 (14.26)	7,913 (16.10)	4,291 (12.86)	886 (9.55)
2012	10,962 (11.94)	7,492 (15.24)	2,965 (8.88)	505 (5.44)
2013	8,874 (9.67)	6,269 (12.75)	2,269 (6.80)	336 (3.62)

^a^Data are presented as number of subjects and percentage.

Distribution for each independent variable had a significant difference among the no antidiabetic drug group, oral antidiabetic group, and with insulin group at a 5% significance level.

The proportion of Medical-aid patients was higher in the group that used insulin (7.71%) than in the groups that used no antidiabetic drugs (5.08%) or oral antidiabetic drugs only (5.89%) (P < 0.0001). The majority of patients with T2DM did not have frequently-visited physicians (85.67%), while approximately half of the patients were diagnosed with T2DM in clinics (55.08%), whereas fewer were diagnosed in general hospitals (12.36%) or general teaching hospitals (32.56%). Over half of the patients (66.82%) were diagnosed with T2DM by internal medicine specialists. Patients who were using insulin had a significantly higher CCI (9.26% in patients with CCI ≥ 4) compared with those in the non-use of antidiabetic drug group (2.56%) and the oral antidiabetic drug group (4.92%) (P < 0.0001). Further analysis showed that the higher CCI score among the Medical-aid group than the higher income group was persistently found, not only among patients in the insulin use group (9.8% vs. 9.2%, P < 0.0001) but also in patients in the oral antidiabetic drug group (5.7% vs. 4.8%, P < 0.0001) and those in the non-use of antidiabetic drug T2DM group (3.7% vs. 2.5%, P < 0.0001).

### Time to start insulin

The time to start insulin use was defined as the period from the day of diagnosis to the day of the first insulin prescription. Table 2 shows the results based on the patient’s health insurance premium and access to a frequently-visited physician. The mean time to starting insulin was 830.47 days overall, although the time decreased as the health insurance premium decreased, falling to a mean time of only 490.66 days in the Medical-aid group. The differences between the groups were significant. Patients with a frequently-visited physician tended to have delayed times to starting insulin (average 864.34 days) compared to those without a frequently-visited physician (average 821.18 days), although this was only marginally significant (P = 0.0935). However, Medical-aid patients without a frequently-visited physician had a significant delay to initiating insulin (average 537.97 days) compared to patients with frequently-visited physicians (average 419.51 days) (P < 0.0001).

**Table 2:** Time to start insulin use in the insulin-treated group.

Group	Number of participants	Duration time to insulin use (days): mean (SD)	P-value
Total	9281	830.47 (987.03)	
Health Insurance premium levels			<0.0001
Medical-aid group	716	490.66 (621.03)	
Levels 1–4	2766	854.96 (987.99)	
Levels 5–7	2546	819.57 (996.21)	
Levels 8 and 9	2095	876.29 (1017.39)	
Level 10	1158	923.10 (1049.33)	
Presence of frequently-visited physicians			0.0935
No	7285	821.18 (991.07)	
Yes	1996	864.34 (971.65)	
Presence of frequently-visited physicians and health insurance premium levels			
With frequently-visited physicians			
Health insurance premium levels			<0.0001
Medical-aid group	286	419.51 (655.70)	
Levels 1–4	2207	838.92 (984.95)	
Levels 5–7	2068	797.86 (992.08)	
Levels 8 and 9	1747	841.30 (999.88)	
Level 10	977	912.09 (1039.76)	
Without frequently-visited physicians			
Health insurance premium levels			<0.0001
Medical-aid group	430	537.97 (592.93)	
Levels 1–4	559	918.30 (998.28)	
Levels 5–7	478	913.50 (1009.58)	
Levels 8 and 9	348	1051.95 (1085.65)	
Level 10	181	982.52 (1100.64)	

### Survival analysis

Survival analysis was performed to assess the relationship between patient characteristics and insulin use (Table 3). Insulin use tended to increase (P < 0.0001) as income decreased. Insulin was used ~2.8 times more often in the Medical-aid group than in the highest premium group (HR: 2.874, 95% CI: 2.588–3.192). Additionally, patients with a frequently-visited physician were more likely to use insulin (HR: 1.549, 95% CI: 1.434–1.624).

**Table 3:** Overall survival analysis^a^.

Independent variable	Dependent variable: Insulin use		
	Hazard ratio	95% confidential interval	P-value
Health insurance premium levels			
Medical-aid group	2.874	2.588–3.192	<0.0001
Levels 1–4	1.485	1.385–1.592	
Levels 5–7	1.338	1.247–1.435	
Levels 8 and 9	1.202	1.118–1.292	
Level 10 (reference)	-	-	
Access to a frequently-visited physicians			
Yes	1.549	1.470–1.631	<0.0001
No (reference)	-	-	
N	91,810		

^a^Other covariates that were controlled for included age, sex, insurance qualification, prescriber characteristics, Charlson comorbidity index, residential area, and year of diagnosis.

Survival analyses were performed for subgroups based on access to frequently-visited physicians and the level of health insurance (Tables 4 and 5). The magnitude of the negative income gradient on the likelihood of insulin use (i.e., the higher likelihood of insulin use, the lower income group) became more apparent in patients who had access to a frequently-visited physician than in those without access (P <0.0001). Patients who received Medical-aid and with access to a frequently-visited physician were ~3.8 times more likely to be prescribed insulin than patients paying the highest premium level (HR: 3.798, 95% CI: 3.094–4.663); in contrast, this difference was 2.3 times higher for patients who received Medical-aid and had no access to a frequently-visited physician (HR: 2.286, 95% CI: 1.986–2.630) (upper panel of Table 4). Survival analysis by health insurance premium also indicated that insulin use was more likely among patients from lower income groups who had access to a frequently-visited physician. In the Medical-aid group, patients with a frequently-visited physician were ~71% more likely to use insulin than those without a frequently-visited physicians (HR: 1.715, 95% CI: 1.461–2.013), accounting for a difference of 41% in the highest premium group (HR: 1.416, 95% CI: 1.203–1.668).

**Table 4:** Subgroup survival analysis based on frequently-visited physicians access^1^.

Independent variable (upper panel)	Dependent variable: insulin use			
	Subgroups by the presence of frequently-visited physicians			
	Frequently-visited physicians (N = 13,152)		No frequently-visited physicians (N = 78,658)	
	HR^a^ (95% CI)^a^	P-value	HR^a^ (95% CI)^a^	P-value
Health insurance premium levels				
Medical-aid	3.798 (3.094–4.663)	<0.0001	2.286 (1.986–2.630)	<0.0001
Levels 1–4	1.567 (1.323–1.857)		1.476 (1.367–1.593)	
Levels 5–7	1.458 (1.227–1.734)		1.317 (1.219–1.422)	
Levels 8 and 9	1.292 (1.079–1.547)		1.185 (1.096–1.282)	
Level 10 (reference)	-		-	

^1^Other covariates that were controlled for included age, sex, insurance qualification, prescriber characteristics, Charlson comorbidity index, residential area, and year of diagnosis.

^a^ HR, hazard ratio; CI, confidence interval.

**Table 5:** Subgroup survival analysis based on health insurance premium^1^.

Independent variable	Subgroups in terms of health insurance premium levels				
	Medical-aid (N = 5,185)	Levels 1–4 (N = 24,823)	Levels 5–7 (N = 24,425)	Levels 8 and 9 (N = 22,739)	Level 10 (N = 14,638)
	HR^a^ (95% CI)^a^	HR^a^ (95% CI)^a^	HR^a^ (95% CI)^a^	HR^a^ (95% CI)^a^	HR^a^ (95% CI)^a^
Presence of frequently-visited physicians					
Yes	1.715 (1.461–2.013)	1.441 (1.311–1.585)	1.509 (1.363–1.671)	1.543 (1.371–1.736)	1.416 (1.203–1.668)
No (reference)	-	-	-	-	-

^1^Other covariates that were controlled for included age, sex, insurance qualification, prescriber characteristics, Charlson comorbidity index, residential area, and year of diagnosis.

^a^ HR, hazard ratio; CI, confidence interval.

## Discussion

We assessed the current state of T2DM management in Korea, focusing on the use of insulin based on NHIS claim data. We also evaluated the differences in insulin use based on the economic status and access to a frequently-visited physician. The results showed that insulin use increased as the health insurance premium decreased. Those with a low income are less likely to be aware of their health conditions as a result of the difficulty obtaining a regular health check-up [18,37–39], therefore, they are more likely to visit clinics or hospitals after they experience symptoms of diabetes. If T2DM management is inadequate at the early stages, it can quickly worsen and potentially increase the rates of early insulin use. As T2DM has already progressed, low-income patients are likely to start insulin after diagnosis. Insulin is used when HbA1c is > 9.0%, or when metformin is ineffective. According to Korea Diabetes Fact Sheet 2018, only 60% of diabetes patients recognized that they had diabetes, and 43.1% patients who had diabetes were not treated in Korea [3]. In Japan, ~ 35% of patients strongly suspected of having diabetes were not treated [40,41]. In addition, the time to insulin use was 1.34 years among all Medical-aid patients and increased to 1.47 years among Medical-aid patients without access to a frequently-visited physician. These were notably shorter than the usual lag time of 10–15 years when using a traditional stepwise strategy [42].

Our finding that insulin use started earlier for patients with a low income is consistent with that of previous reports. A study in the United Kingdom showed that multi-morbidities, including diabetes, developed 10–15 years earlier in regions with the lowest incomes compared with regions with the highest incomes [18]. A systematic review also showed that low income is associated with an increased risk of T2DM [43]. Several studies in Asian countries have reported similar findings. For example, a study in Hong Kong showed that the lower income group had a higher risk of diabetes and poorer prognosis than the higher income group [44]. A study in China also showed that patients with a lower income had a higher incidence of diabetic retinopathy and neuropathy [45], indicating either poor or non-timely management of diabetes. A study in Taiwan using the National Health Insurance Research Database also estimated that the incidence of T2DM was higher in the those with a lower income, who were also less likely to visit diabetes clinics [46]. Studies in Japan have also reported that those with a low income had a higher incidence of T2DM and higher T2DM treatment rate [26,47].

Another interesting finding in the present study was that the majority of patients did not have a frequently-visited physician. It may be that frequently-visited physician system has not been legislated in Korea, resulting in a lack of familiarity of the concept of frequently-visited physicians with no enforcement. The likelihood of insulin use was also higher in the Medical-aid group when patients had frequently-visited physicians than when they did not in the present study, which ironically indicated that access to a frequently-visited physician at the time of T2DM diagnosis improves management of the condition. Using insulin may be dangerous without appropriate vigilance as it can lead to severe hypoglycemia [48]. Therefore, frequently-visited physicians could base care for T2DM on the clinical status of their patients in a more timely manner [49], starting insulin use without necessarily offering oral antidiabetic drugs if their clinical diagnosis shows progression of T2DM warranting insulin [5]. Consistent with this, a cross-sectional online survey showed that > 80% of insulin use was initiated by primary physicians [50]. Communication between physician and patients can improve adherence to medication [51], thus frequent interactions between the patient and the frequently-visited physicians can reduce the reluctance to use insulin. Taken together, these findings suggest that T2DM among low-income patients can be managed more effectively with constant support by a frequently-visited physician.

The positive effects of appropriate management by primary physicians have been investigated in terms of the outcomes and prevention of complications among patients with chronic diseases, including diabetes [28–30]. The quality of medical care for patients with T2DM improved as the ratio of primary physicians increased [28], while patients with T2DM who had primary physicians also experienced improved outcomes following intensive medication and lifestyle counseling [29]. Several studies have also shown improved glycemic control when diabetic patients were under continuous care, and this continuous care was better with access to a primary care physicians [52,53]. Additionally, primary care physicians provide superior care though lifestyle counseling and improved medical treatment [29], and frequent visits to a physician have been shown to achieve HbA1c targets faster in patients with diabetes [54].

However, few studies have identified the impact of care by primary physicians on patient outcomes in Asia, where the system for primary care is not yet well established [55,56]. Previous studies in Korea and Japan have reported that patients consider specialists and larger hospitals to provide better services and outcomes, despite the extra cost and effort [55,57,58]. In the present study, primary physicians were defined on the basis of visit frequency in the absence of clear distinction in Korea. When patients with T2DM attended clinics more regularly to see a frequently-visited physician, the timeliness of management decisions may have been improved. There is a ‘Chronic Disease Management System in Clinics’ in Korea, in which the NHIS reduces the coinsurance rate of the out-of-pocket cost from 30% to 20% if patients with T2DM designate a particular clinic for long-term management [59]. By encouraging patients to visit the clinic regularly, early diagnosis and treatment of diabetes is made easier. Such persistent management of T2DM regularly by a gate-keeping physician could reduce cardiovascular risks, blindness, kidney failure, amputation, and depression [60,61]. Therefore, it is necessary to intensify check-ups and increase the education of patients who already have diabetes based on the results. Currently, the Korean Diabetes Association is also advocating the benefits of insurance through counseling for diabetes education and expansion of the diabetes prevention program [62]. Our results offer real-world evidence to support the effectiveness and importance of regular management of T2DM, as the Korean Diabetes Association and Korean NHIS promote.

There are a number of strengths to the present study. First, our estimations were based on the claims data of the NHIS, which is a public health insurance that covers all legal residents of Korea. Therefore, our results are considered to be nationally representative. Second, we focused on the insulin use of newly diagnosed patients, which could implicitly indicate the extent of T2DM advancement. We did not include insulin use in inpatients admitted for emergency or ward care, making it possible to exclude shock and other emergency situations that can be accompanied by increased blood sugar levels requiring insulin treatment [63]. However, we acknowledge several caveats of the present study. First, uninsured medications were not included in NHIS claims despite the majority of antidiabetic drugs being covered by the NHIS [64]. Second, neither the true severity of T2DM nor whether patients took medicines can be accurately measured on the basis of insurance claims data.

Cohort data with detailed medical information on T2DM and patient socioeconomic characteristics would advance current understanding on diabetes treatment patterns by patients’ socioeconomic conditions. Further information on the patient-physician relationship, including the duration of the relationship, would assist in establishing an effective primary care physician system, particularly where such system is not currently in place. In addition, the relationship between the frequency of regular check-ups and the management of T2DM may provide evidence on the importance of regular check-ups.

## Conclusions

Patients with low levels of income, as defined based on their level of health insurance, were more likely to use insulin and to have a short lag time before insulin therapy was started. The likelihood of insulin use was higher if a patient had access to a frequently-visited physician than if they did not, particularly if they also had a low income. Therefore, we conclude that a patient’s economic status requires consideration when seeking to provide effective treatment for T2DM. Utilizing frequently-visited physicians might improve the management of this chronic disease, particularly among patients with a low income.

## References

[1] WHO. Global report on diabetes. Geneva: World Health Organization Available from: http://apps.who.int/medicinedocs/en/m/abstract/Js22371en/.

[2] IDF DIABETES ATLAS 2017. International Diabetes Federation. Available from: http://diabetesatlas.org/IDF_Diabetes_Atlas_8e_interactive_EN/.

[3] Diabetes fact sheet in Korea 2018. Korea Diabetes Association. Available from: http://www.diabetes.or.kr/pro/news/admin.php?category=A&code=admin&number=1546&mode=view.

[4] Diabetes fact sheet in Korea 2015. Korea Diabetes Association. Available from: http://www.diabetes.or.kr/pro/news/admin.php?category=B&code=admin&mode=view&number=1427.

[5] Standards of medical care in diabetes-2017. American Diabetes Association. Available from: http://care.diabetesjournals.org/content/diacare/suppl/2016/12/15/40.Supplement_1.DC1/DC_40_S1_final.pdf. 15618112

[6] Alsahli M, Gerich JE. Hypoglycemia, chronic kidney disease, and diabetes mellitus. Mayo Clin Proc. 2014;89: 1564–1571. 10.1016/j.mayocp.2014.07.013 25305751

[7] KDIGO 2012 Clinical Practice Guideline for the Evaluation and Management of Chronic Kidney Disease. Kidney Disease: Improving Global Outcomes Chronic Kidney Disease Guideline Development Work Group. International Society of Nephrology. 2013;3.

[8] Apelqvist J, Bakker K, van Houtum WH, Schaper NC, International Working Group on the Diabetic Foot Editorial B. Practical guidelines on the management and prevention of the diabetic foot: based upon the International Consensus on the Diabetic Foot (2007) Prepared by the International Working Group on the Diabetic Foot. Diabetes Metab Res Rev. 2008;24 Suppl 1: S181–S187. 18442189 10.1002/dmrr.848

[9] Singh R, Kishore L, Kaur N. Diabetic peripheral neuropathy: current perspective and future directions. Pharmacol Res. 2014;80: 21–35. 10.1016/j.phrs.2013.12.005 24373831

[10] Vinik AI, Nevoret ML, Casellini C, Parson H. Diabetic neuropathy. Endocrinol Metab Clin North Am. 2013;42: 747–787. 10.1016/j.ecl.2013.06.001 24286949

[11] Treatment Guidelines for Diabetes (5th edition). Korean Diabtes Association. Available from: http://www.diabetes.or.kr/pro/publish/guide.php?code=guide&year_v=2015&number=638&mode=view.

[12] Treatment guidelines for diabetes in Korea. Korean Diabetes Association. Available from: http://www.diabetes.or.kr/pro/publish/guide.php?code=guide&number=638&mode=view.

[13] Authors/Task Force M, Ryden L, Grant PJ, Anker SD, Berne C, Cosentino F, et al ESC Guidelines on diabetes, pre-diabetes, and cardiovascular diseases developed in collaboration with the EASD: the Task Force on diabetes, pre-diabetes, and cardiovascular diseases of the European Society of Cardiology (ESC) and developed in collaboration with the European Association for the Study of Diabetes (EASD). Eur Heart J. 2013;34: 3035–3087. 10.1093/eurheartj/eht108 23996285

[14] Global guideline for type 2 diabetes. International Diabetes Federation Guideline Development Group. Diabetes Res Clin Pract. 2014;104: 1–52. 10.1016/j.diabres.2012.10.001 24508150

[15] Pharmacologic Treatment Guidelines for Diabetes. Korean Diabtes Association. Available from: http://www.diabetes.or.kr/pro/publish/guide.php?code=guide&year_v=2017&number=672&mode=view.

[16] Gavin JR 3rd, Freeman JS, Shubrook JH Jr., Lavernia F. Type 2 diabetes mellitus: practical approaches for primary care physicians. J Am Osteopath Assoc. 2011;111: S3–S12; quiz S13. 21697547

[17] National Diabetes Statistics Report, 2017. Centers for Disease Control and Prevention, US Department of Health and Human Services. Available from: https://www.cdc.gov/diabetes/pdfs/data/statistics/national-diabetes-statistics-report.pdf.

[18] Barnett K, Mercer SW, Norbury M, Watt G, Wyke S, Guthrie B. Epidemiology of multimorbidity and implications for health care, research, and medical education: a cross-sectional study. Lancet. 2012;380: 37–43. 10.1016/S0140-6736(12)60240-2 22579043

[19] Lasser KE, Himmelstein DU, Woolhandler S. Access to care, health status, and health disparities in the United States and Canada: results of a cross-national population-based survey. Am J Public Health. 2006;96: 1300–1307. 10.2105/AJPH.2004.059402 16735628 PMC1483879

[20] Loignon C, Hudon C, Goulet E, Boyer S, De Laat M, Fournier N, et al Perceived barriers to healthcare for persons living in poverty in Quebec, Canada: the EQUIhealThY project. Int J Equity Health. 2015;14: 4 10.1186/s12939-015-0135-5 25596816 PMC4300157

[21] Weinick RM, Byron SC, Bierman AS. Who can't pay for health care? J Gen Intern Med. 2005;20: 504–509. 10.1111/j.1525-1497.2005.0087.x 15987324 PMC1490134

[22] Ko B, Lim J, Kim YZ, Park HS. Trends in type 2 diabetes prevalence according to income levels in Korea (1998–2012). Diabetes Res Clin Pract. 2016;115: 137–139. 10.1016/j.diabres.2016.01.014 26830075

[23] Kim SR, Han K, Choi JY, Ersek J, Liu J, Jo SJ, et al Age- and sex-specific relationships between household income, education, and diabetes mellitus in Korean adults: the Korea National Health and Nutrition Examination Survey, 2008–2010. PLoS One. 2015;10: e0117034 10.1371/journal.pone.0117034 25622031 PMC4306546

[24] Hwang J, Shon C. Relationship between socioeconomic status and type 2 diabetes: results from Korea National Health and Nutrition Examination Survey (KNHANES) 2010–2012. BMJ Open. 2014;4: e005710 10.1136/bmjopen-2014-005710 25138810 PMC4139629

[25] Kindo B, Himanshu R, Parmar K, Dube S, Ramesh J. Socioeconomic and demographic trends in the prevalence of type 2 diabetes in India. J Soc Health Diabetes. 2016;4: 90–101.

[26] Hayashino Y, Yamazaki S, Nakayama T, Sokejima S, Fukuhara S. The association between socioeconomic status and prevalence of diabetes mellitus in rural Japan. Arch Environ Occup Health. 2010;65: 224–229. 10.1080/19338244.2010.486423 21186428

[27] Wu H, Meng X, Wild SH, Gasevic D, Jackson CA. Socioeconomic status and prevalence of type 2 diabetes in mainland China, Hong Kong and Taiwan: a systematic review. J Glob Health. 2017;7: 011103 10.7189/jogh.07.011103 28702177 PMC5481892

[28] Furno M. The primary role: how the availability of primary care physicians affects diabetes care management. J Med Pract Manage. 2014;30: 139–143. 25807609

[29] Morrison F, Shubina M, Goldberg SI, Turchin A. Performance of primary care physicians and other providers on key process measures in the treatment of diabetes. Diabetes Care. 2013;36: 1147–1152. 10.2337/dc12-1382 23230095 PMC3631881

[30] Clement FM, Chen G, Khan N, Tu K, Campbell NR, Smith M, et al Primary care physician visits by patients with incident hypertension. Can J Cardiol. 2014;30: 653–660. 10.1016/j.cjca.2014.03.033 24882537

[31] Chelimsky TC, Fischer RL, Levin JB, Cheren MI, Marsh SK, Janata JW. The primary practice physician program for chronic pain ((c) 4PCP): outcomes of a primary physician-pain specialist collaboration for community-based training and support. Clin J Pain. 2013;29: 1036–1043. 10.1097/AJP.0b013e3182851584 23459398

[32] Weng W, Liang Y, Kimball ES, Hobbs T, Kong S, Sakurada B, et al Drug usage patterns and treatment costs in newly-diagnosed type 2 diabetes mellitus cases, 2007 vs 2012: findings from a large US healthcare claims database analysis. J Med Econ. 2016;19: 655–662. 10.3111/13696998.2016.1151795 26855139

[33] Kalsekar ID, Madhavan SS, Amonkar MM, Douglas SM, Makela E, Elswick BL, et al Impact of depression on utilization patterns of oral hypoglycemic agents in patients newly diagnosed with type 2 diabetes mellitus: a retrospective cohort analysis. Clin Ther. 2006;28: 306–318. 10.1016/j.clinthera.2006.02.005 16678652

[34] Regulation for Criteria for Providing Reimbursed Services in the National Health Insurance. Ministry of Health and Walfare. Available from: http://www.law.go.kr/%EB%B2%95%EB%A0%B9/%EA%B5%AD%EB%AF%BC%EA%B1%B4%EA%B0%95%EB%B3%B4%ED%97%98%EC%9A%94%EC%96%91%EA%B8%89%EC%97%AC%EC%9D%98%EA%B8%B0%EC%A4%80%EC%97%90%EA%B4%80%ED%95%9C%EA%B7%9C%EC%B9%99.

[35] 2018 Medical Care Service. Ministry of Health and Walfare. Available from: http://www.bokjiro.go.kr/welInfo/retrieveGvmtWelInfo.do?welInfSno=310.

[36] Charlson ME, Pompei P, Ales KL, MacKenzie CR. A new method of classifying prognostic comorbidity in longitudinal studies: development and validation. J Chronic Dis. 1987;40: 373–383. 3558716 10.1016/0021-9681(87)90171-8

[37] Churchill CF, Matul M, Rückversicherungs-Gesellschaft M, Microinsurance CGtAtPWGo, Office IL Protecting the poor: a microinsurance compendium: International Labour Office; 2006.

[38] Wong HZ, Lim WY, Ma SS, Chua LA, Heng DM. Health screening behaviour among Singaporeans. Ann Acad Med Singapore. 2015;44: 326–334. 26584661

[39] Kullgren JT, Galbraith AA, Hinrichsen VL, Miroshnik I, Penfold RB, Rosenthal MB, et al Health care use and decision making among lower-income families in high-deductible health plans. Arch Intern Med. 2010;170: 1918–1925. 10.1001/archinternmed.2010.428 21098352 PMC4004054

[40] Goto M, Goto A, Ikeda N, Noda H, Shibuya K, Noda M. Factors associated with untreated diabetes: analysis of data from 20,496 participants in the Japanese National Health and Nutrition Survey. PLoS One. 2015;10: e0118749 10.1371/journal.pone.0118749 25756183 PMC4355906

[41] Reference data of Healthy Japan 21 (Second campaign) (in Japanese). Ministry of Health, Labour and Welfare. Available from: http://www.mhlw.go.jp/bunya/kenkou/dl/kenkounippon21_02.pdf.

[42] Nathan DM. Clinical practice. Initial management of glycemia in type 2 diabetes mellitus. N Engl J Med. 2002;347: 1342–1349. 10.1056/NEJMcp021106 12397193

[43] Agardh E, Allebeck P, Hallqvist J, Moradi T, Sidorchuk A. Type 2 diabetes incidence and socio-economic position: a systematic review and meta-analysis. Int J Epidemiol. 2011;40: 804–818. 10.1093/ije/dyr029 21335614

[44] Wong MC, Leung MC, Tsang CS, Lo SV, Griffiths SM. The rising tide of diabetes mellitus in a Chinese population: a population-based household survey on 121,895 persons. Int J Public Health. 2013;58: 269–276. 10.1007/s00038-012-0364-y 22552749

[45] Emoto N, Okajima F, Sugihara H, Goto R. A socioeconomic and behavioral survey of patients with difficult-to-control type 2 diabetes mellitus reveals an association between diabetic retinopathy and educational attainment. Patient Prefer Adherence. 2016;10: 2151–2162. 10.2147/PPA.S116198 27822016 PMC5087708

[46] Hsu CC, Lee CH, Wahlqvist ML, Huang HL, Chang HY, Chen L, et al Poverty increases type 2 diabetes incidence and inequality of care despite universal health coverage. Diabetes Care. 2012;35: 2286–2292. 10.2337/dc11-2052 22912425 PMC3476930

[47] Fukuda Y, Hiyoshi A. Association of income with symptoms, morbidities and healthcare usage among Japanese adults. Environ Health Prev Med. 2012;17: 299–306. 10.1007/s12199-011-0254-6 22180347 PMC3390568

[48] Liu J, Wang R, Ganz ML, Paprocki Y, Schneider D, Weatherall J. The burden of severe hypoglycemia in type 2 diabetes. Curr Med Res Opin. 2018;34: 179–186. 10.1080/03007995.2017.1391080 29017368

[49] Emerson S. Implementing diabetes self-management education in primary care. Diabetes Spectrum. 2006;19: 79–83.

[50] Kalirai S, Stephenson J, Perez-Nieves M, Grabner M, Hadjiyianni I, Geremakis C, et al Primary care physician perspectives on basal insulin initiation and maintenance in patients with type 2 diabetes mellitus. Prim Care Diabetes. 2017 10.1016/j.pcd.2017.10.001 29100717

[51] Zolnierek KB, Dimatteo MR. Physician communication and patient adherence to treatment: a meta-analysis. Med Care. 2009;47: 826–834. 10.1097/MLR.0b013e31819a5acc 19584762 PMC2728700

[52] Mainous AG 3rd, Koopman RJ, Gill JM, Baker R, Pearson WS. Relationship between continuity of care and diabetes control: evidence from the Third National Health and Nutrition Examination Survey. Am J Public Health. 2004;94: 66–70. 14713700 10.2105/ajph.94.1.66 PMC1449828

[53] Dearinger AT, Wilson JF, Griffith CH, Scutchfield FD. The effect of physician continuity on diabetic outcomes in a resident continuity clinic. J Gen Intern Med. 2008;23: 937–941. 10.1007/s11606-008-0654-5 18612720 PMC2517915

[54] Morrison F, Shubina M, Turchin A. Encounter frequency and serum glucose level, blood pressure, and cholesterol level control in patients with diabetes mellitus. Arch Intern Med. 2011;171: 1542–1550. 10.1001/archinternmed.2011.400 21949161 PMC3692291

[55] Osug Y, Deshpande GA, Takahashi O, Arioka H, Ino T, Asai K, et al Impact of primary care physicians on hospital mortality, readmission rate, and length of stay in Japanese Healthcare System. Quality Prim Care. 2016;24: 298–302.

[56] Chan TK, Lee CY, Yau SK, Tipoe GL. Primary care in Asia: a call for compulsory vocational training. Br J Gen Pract. 2014;64: e381–e383. 10.3399/bjgp14X680281 24868076 PMC4032021

[57] Ock M, Kim JE, Jo MW, Lee HJ, Kim HJ, Lee JY. Perceptions of primary care in Korea: a comparison of patient and physician focus group discussions. BMC Fam Pract. 2014;15: 178 10.1186/s12875-014-0178-5 25358391 PMC4236417

[58] Kim Y, Kim J. A study on healthcare institution selection of healthcare consumers using theory of consumption values: focusing on relations among clinics or small sized hospitals, general hospitals, and large-sized hospitals. J Korean Soc Qual Manag. 2009;37: 71–86.

[59] Chronic Disease Management System at Clinics. National Health Insurance Service. Available from: http://hi.nhis.or.kr/bc/ggpbc001/ggpbc001_m01.do.

[60] Herman WH, Ye W, Griffin SJ, Simmons RK, Davies MJ, Khunti K, et al Early detection and treatment of type 2 diabetes reduce cardiovascular morbidity and mortality: a simulation of the results of the Anglo-Danish-Dutch study of intensive treatment in people with screen-detected diabetes in primary care (ADDITION-Europe). Diabetes Care. 2015;38: 1449–1455. 10.2337/dc14-2459 25986661 PMC4512138

[61] Diabetes: The Silent Pandemic and its Impact on Australia. Baker IDI Heart & Diabetes Institute 2012.

[62] Proposal of Korean Diabetes Prevention Management Policy and Strategy. Korean Diabetes Association. Available from: http://www.diabetes.or.kr/pro/news/sub03.php?code=brief&number=642&mode=view.

[63] Glynn N, Owens L, Bennett K, Healy ML, Silke B. Glucose as a risk predictor in acute medical emergency admissions. Diabetes Res Clin Pract. 2014;103: 119–126. 10.1016/j.diabres.2013.10.015 24269157

[64] [General Principles] Diabetic Medication. Available from: https://www.hira.or.kr/rd/insuadtcrtr/InsuAdtCrtrPopup.do?mtgHmeDd=20171001&sno=1&mtgMtrRegSno=0017.

